# Local Infiltrations in Patients with Radiculopathy or Chronic Low Back Pain Due to Segment Degeneration—Only A Diagnostic Value?

**DOI:** 10.3390/jpm12111791

**Published:** 2022-10-30

**Authors:** Chris Lindemann, Timo Zippelius, Felix Hochberger, Alexander Hölzl, Sabrina Böhle, Patrick Strube

**Affiliations:** 1 Orthopedic Department, Jena University Hospital, Campus Eisenberg, 07607 Eisenberg, Germany; 2 Department of Orthopedic Surgery, University of Ulm, 89081 Ulm, Germany; 3 Department of Orthopedic Sports Medicine, Klinikum Rechts der Isar, Technical University of Munich, 81675 Munich, Germany

**Keywords:** facet joint capsule infiltration, periradicular infiltration, selective nerve block, radiculopathy, low back pain

## Abstract

The purpose of this study was to investigate the differences in the therapeutic effectiveness of CT-assisted infiltration of a local anesthetic + corticosteroid between nerve root and facet joint capsule in patients with chronic complaints. In this prospective trial with a 12-month follow-up, a total of 250 patients with chronic low back pain and radiculopathy were assigned to two groups. In the first group, patients with specific lumbar pain due to spondyloarthritis received periarticular facet joint capsule infiltration (FJI). In the second group, patients with monoradicular pain received periradicular infiltration (PRI) via an extraforaminal selective nerve block. Clinical improvement after FJI and PRI regarding pain (NRS), function (ODI), satisfaction (McNab), and health related quality of life (SF-36) were compared. Minimally clinically important difference (MCID) served as the threshold for therapeutic effectiveness evaluation. A total of 196 patients were available for final analysis. With respect to the pain reduction and functional improvement (ODI, NRSoverall, and NRSback), the PRI group performed significantly better (ptreatment < 0.001) and longer over time (ptreatment × time 0.001) than the FJI group. Regarding pain and function, only PRI demonstrated a durable improvement larger than MCID. A significant and durable therapeutic value was found only after receiving PRI but not after FJI in patients with chronic pain.

## 1. Introduction

Degenerative changes in the lumbar spine are a common cause of chronic pain, physical limitations, reduced health-related quality of life, and absenteeism. As a consequence, these changes are associated with considerable social and health costs in Western societies [1,2,3,4,5].

Facet joint degeneration, a real joint-segment-joint degeneration, is often a trigger of back pain. In addition, disc herniation, recess, or neuroforaminal stenosis often affects the nerve root and can lead to leg pain [6,7]. A variety of treatment methods are available for chronic diseases due to lumbar segment degeneration. Among these treatments, local infiltration therapy also holds differential diagnostic value.

Local anesthetics and steroids are often used here. Local anesthetics have been postulated to provide relief by various mechanisms, i.e., suppression of nociceptive discharge, the block of the sympathetic reflex arc, the blockade of the axonal transport, and anti-inflammatory effects [8]. Steroids act via two mechanisms. On one hand, steroids have anti-inflammatory, anti-edematous, and immunosuppressive properties. On the other hand, steroids inhibit neuronal transmission within C-fibers, helping to reduce both lumbar and radicular pain symptoms [6,8,9]. Furthermore, Computed Tomography (CT)-supported infiltration represents a precise and reproducible therapy option associated with reduced complication rates [10,11,12,13].

However, based on current research, the duration of the effect of therapeutically intended infiltration as it relates to the treated tissue, nerve root, or facet joint, particularly in the case of chronic and specific complaints, remains unclear. In previous studies, infiltration locations were considered partially together and partially individually compared with placebo groups, and the temporal aspect of complaint duration was insufficient [14]. However, given the different associated pathoanatomical processes (e.g., arthrosis vs. neuronal compression), differences are likely. Therefore, the aim of this study was to investigate the therapeutic value of CT-based infiltration in terms of pain, function, and quality of life based on the kind of injection (periradicular infiltration, PRI vs. facet joint capsule infiltration, FJI) in chronic complaints. We hypothesized that FJI has a shorter duration of effectiveness and therefore less therapeutic value than PRI.

## 2. Materials and Methods

### 2.1. Study Design

A total of 250 patients were screened in a single-center (university hospital, orthopedic department), prospective, nonrandomized, and nonblinded study between June 2018 and December 2019. Patients were included in the event of their consent approval. The number of cases was calculated based on the statistical parameters of the clinical outcome scores of a test cohort. The effect size for the power analysis based on a 2-sided, 2-way ANOVA with 4 or 5 measurement repetitions, and was thus set to 0.12. With a ß of 0.2 and an α of 0.05, the required group size was determined to be 86 patients. Assuming a rather conservative calculated dropout rate in telephone interviews of 45% after one year, the total group size was approximately 125 patients per group. The presented study was registered with the Trial Registry Number: DRKS00023722 (German Registry of Clinical Trials; 8 December 2020).

### 2.2. Ethical Approval

Study approval was obtained from the ethics committee of the University Hospital Jena, Germany (No. 5487-3/18) and all methods were performed in accordance with the relevant guidelines and regulations. All patients were informed about the study preinterventionally and gave their written informed consent to participate in the study.

### 2.3. Patients and Groups

The inclusion criteria were patients aged ≥ 18 years with predominantly low back pain or predominantly monoradicular leg pain after the failure of structured noninvasive conservative treatment with pain relievers and physiotherapy for at least six weeks and a complaint duration of at least 12 weeks. This includes exercise, paracetamol or Nonsteroidal Anti-inflammatory Drugs, manual therapy, acupuncture, and spinal manipulation in patients with radiculopathy. PRI was performed in patients with predominantly unilateral lumbar radiculopathy based on single-level nerve root compression (caused by a herniated disc, stenosis of the lateral recess or neuroforamen) confirmed by morphological imaging (MRI or CT). FJI was performed in patients with predominantly specific lumbar pain due to single-level lumbar segment degeneration (Fujiwara grade ≥ 3° spondyloarthritis with partial additional intervertebral disc degeneration, osteochondrosis, degenerative spondylolisthesis) confirmed by morphological imaging (MRI or CT) [15]. The definition for predominant pain resulted from the highest NRS value (back vs. leg). Even if this was similar in a few cases (e.g., NRS leg 6, NRS back 5), the infiltration, if MR morphologically comprehensible, was performed at the predominantly pain-inducing site. All patients were mentally and physically capable of providing consent and processing the questionnaires. During follow-up, included patients were able to receive structured conservative therapy using analgesics and physical therapy.

The exclusion criteria included previous surgeries on the affected spine segment, multilevel pathologies in the MRI of the lumbar spine, and bilateral radicular complaints. Furthermore, patients in whom the peri-interventional risk profile was increased due to other diseases were excluded. These diseases included insufficiently controlled diabetes mellitus, intake of oral anticoagulants, clotting disorders, increased laboratory infection parameters (leukocytosis and increased C-reactive protein, and known infections and/or cancer diseases. Patients who had an absolute surgical indication due to acute serious neurological deficits (e.g., paresis > 3/5 according to Janda and conus/cauda syndrome) were also excluded from the study. In addition, patients who could not meet the requirements for telephone interviews and patients with a known allergy to local anesthetics (LA) or corticosteroids were excluded from the study. Patients were consecutively assigned into two groups depending on the site of infiltration. Patients with lumbar pain due to facet joint arthrosis received FJI (FJI group), whereas patients with radicular pain due to nerve root affection received a PRI (PRI group).

### 2.4. Intervention

All patients were placed prone on the table for the computer tomography scanner (BrightSpeed, Manuf. GE Healthcare) and treated under sterile conditions. All interventions were standardized by a single doctor (CL). Pre-interventional oral drug sedation of the patient was performed as needed.

**Figure 1:**
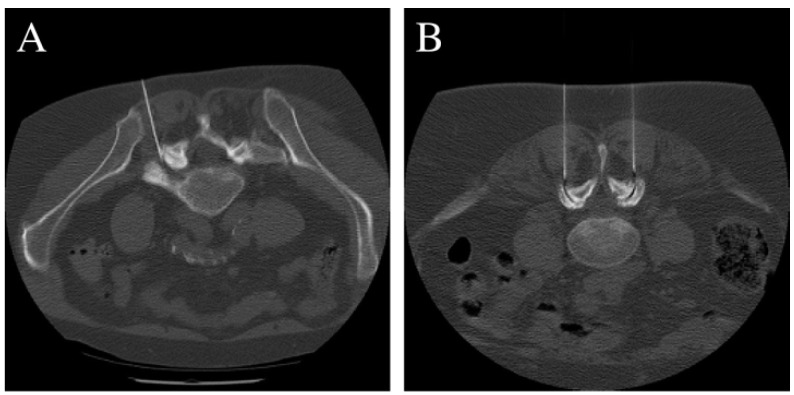
Computed tomography showing the positioning of the spinal needle(s) lateral to the midline at the level of the affected nerve root via an extraforaminal approach at the L5/S1 neuroforamen (**A**) and L4/5 facet joint (**B**).

During FJI treatment, the needles were positioned (2× disposable cannula 1 × 120 mm, Manuf. TSK LABORATORY) after appropriate CT-based identification. As intra-articular injection is often not possible due to advanced degeneration of the facet joints, the needles were placed directly around the affected facet joint capsules (joint line, Figure 1). With regard to the inclusion criteria (single-level pathologies), only the affected facet joint pair was infiltrated. With PRI, the needles were positioned lateral to the midline at the level of the affected nerve root via an extraforaminal approach (analogous to a selective nerve root block). This prevented penetration into the epidural space, and the medication was rinsed around the affected nerve root (Figure 1). The medications used included 1.5 mL local anesthetic (1% Xylocitin^®®^, MIBE GmbH Arzneimittel; Lidocaine hydrochloride) + 0.5 mL corticosteroid (Lipotalon^®®^, Recordati Industria Chimica e Farmaceutica SpA; Dexamethasone) or only 2 mL LA in cases of steroid allergy/intolerance. No contrast agent was used in either group due to CT-secured needle positioning. Before injection, needle aspiration was performed to prevent vascular spread.

### 2.5. Epidemiological and Clinical Data

When patients were first assessed, data, such as sex, age, weight, height, and body mass index (BMI), were recorded. In addition, grades of facet joint arthritis were determined in the FJI group using the Fujiwara classification system. In the PRI group, MR morphological differentiation of nerve root compression into moderate and advanced was performed [15].

The patient’s overall pain perception was assessed using a numerical rating scale (NRSoverall 0—no pain, 10—maximum pain). Leg pain (NRSleg) and back pain (NRSback) were also assessed in isolation using the same numerical rating scale. The Oswestry Disability Index (ODI) was used to assess functional restriction [16]. The Health Short Form 36 (SF-36) was used to assess the restriction of the health-related quality of life (HrQoL) [17,18,19] and included evaluation of the physical (pcs) and mental (mcs) total scores. Satisfaction with the treatment was assessed using the MacNab criteria [20], with four levels of categorization: excellent, good, fair, and poor.

Pain and function (NRSoverall, NRSback, NRSleg, ODI) were assessed preprocedure and by telephone (except on day one with only NRS score) at the follow-up appointments at 6 weeks and at 3, 6, and 12 months. The SF-36 form was collected preprocedure and queried postprocedure for the 12-month follow-up.

### 2.6. Analysis of the Therapeutic Value

Epidemiological data and patient scores were compared between the groups. To assess the therapeutic value of PRI and FJI, the improvements in the overall pain scale and the ODI compared to the preinterventional value (deltaNRSoverall and deltaODI) were compared to the minimal clinically important difference (MCID) for chronic complaints. According to previous work, the MCID was established for pain deltaNRSoverall = 2. For function, the deltaODI = 16% [21,22,23,24].

### 2.7. Statistics

The statistical evaluation of this work was performed using SPSS Statistics (Version 24, IBM, Armonk, NY, USA).

The demographic data were assessed using Student’s *t*-test for independent samples, and the normal distribution of the data was assessed in advance using the Kolmogorov–Smirnov test. Categorical data were evaluated using Fisher’s exact test, and continuous data were evaluated using Student’s *t*-test. Given that the primary and secondary target values were measured at 5 or 6 points in time, the scores were subjected to a 2-way ANOVA for repeated measures using post hoc Bonferroni tests. The Greenhouse–Geisser correction was used to assess the sphericity. A double-sided significance check was performed for all tests, and a *p*-value < 0.05 was assumed to indicate statistical significance for all statistical tests.

## 3. Results

### 3.1. Baseline Demographics

**Figure 2:**
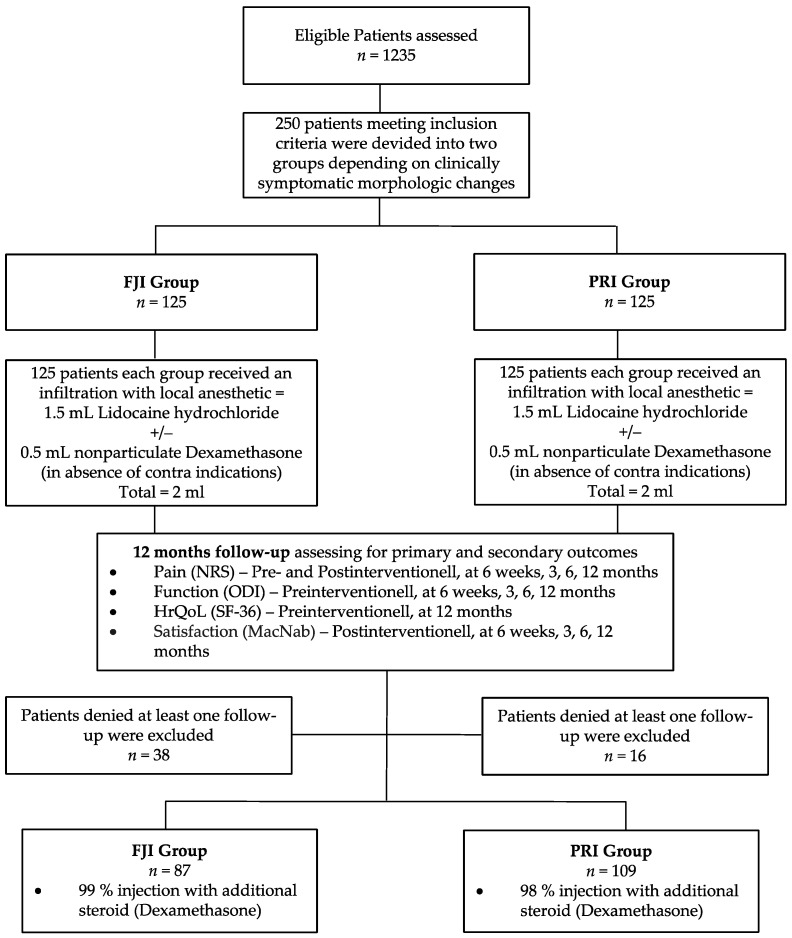
Flow chart. Schematic presentation of participant flow at the 12-month follow-up. FJI—facet joint capsule infiltration; PRI—periradicular infiltration therapy.

A total of 196 patients were available for data analysis (Figure 2). The patient baseline demographics are listed in Table 1. In the FJI group, 39 patients (45%) had infiltrated facet joints L4/5, and 48 patients (55%) had infiltrated facet joints L5/S1. In the PRI group, infiltration of the L3 nerve root occurred in 18 patients (17%), infiltration of the L4 nerve root occurred in 24 patients (22%), infiltration of the L5 nerve root occurred in 56 (51%), and infiltration of the S1 nerve root occurred in 11 patients (10%). According to the study protocol, 86 patients (99%) in the FJI group and 107 patients (98%) in the PRI group were administered an additional steroid (*p* = 0.151).

### 3.2. Results of Pain and Functional Improvement

All clinical scores (ODI, NRSoverall, NRSback, and NRSleg) were significantly improved over time (ptime < 0.001). With respect to the ODI, NRSoverall, and NRSback, the PRI group performed significantly better (ptreatment < 0.001) and longer over time (ptreatment × time 0.001) than the FJI group. Additionally, leg pain was significantly different between the two groups over time (ptreatment < 0.001; ptreatment × time 0.001). For detailed results and post hoc tests, see Table 2 and Table 3.

### 3.3. Results Regarding the Therapeutic Value

**Figure 3:**
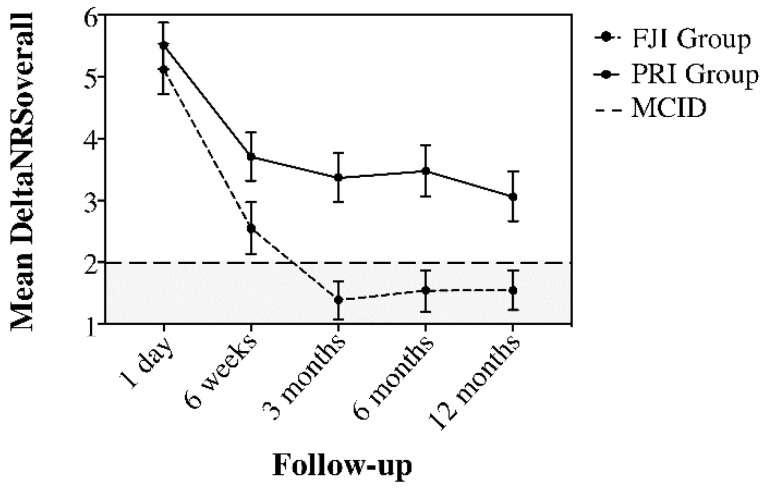
Comparison of deltaNRSoverall between the FJI and PRI groups over the follow-up period to illustrate the duration of the treatment effect concerning the minimal clinically important difference (MCID), (horizontal line at a DeltaNRS of 2). The FJI group fell below the horizontal line in the gray area at the 3-month follow-up, whereas the PRI group presented clinically important improvement (white area) over the complete one-year follow-up. Whiskers represent the 95% confidence interval (CI).

**Figure 4:**
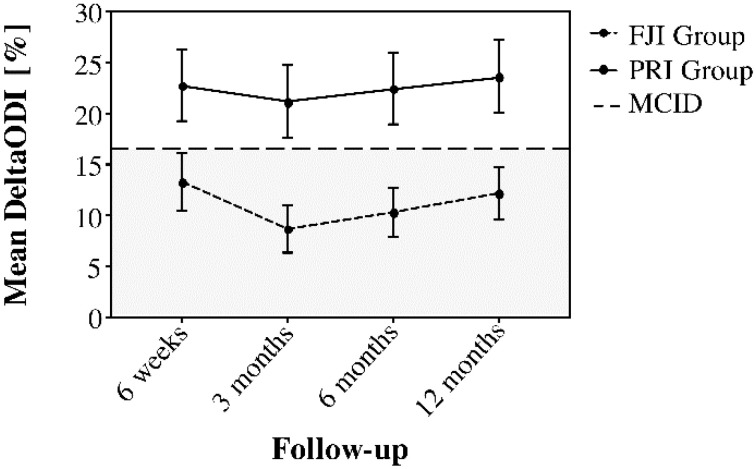
Comparison of deltaODI between the FJI and PRI groups over the follow-up period to illustrate the duration of the treatment effect concerning the minimal clinically important difference (MCID, horizontal line at a deltaODI of 16%). The FJI group was below the horizontal line in the gray area at the 3-month follow-up, whereas the PRI group presented clinically important improvement (white area) over the complete one-year follow-up. Whiskers represent the 95% confidence interval (CI).

Figure 3 and Figure 4 demonstrate the improvement of pain (deltaNRSoverall) and function (deltaODI) in relation to the MCID. Here, the deltaNRSoverall failed to indicate clinical improvement in the FJI group from three months postintervention onwards, whereas the PRI group presented with clinically important improvement over the complete follow-up period. Moreover, the deltaODI of the FJI group never reached the MCID, whereas the ODI improvement of the PRI group was always greater than that of the MCID group.

### 3.4. Health-Related Quality of Life and Patient Satisfaction

Table 4 shows the results of the SF-36. Both groups showed similar baseline pcs values before the intervention. In contrast, patients from the FJI group showed significant deterioration in pcs (*p* = 0.033) after the intervention, whereas the PRI group showed a significant improvement in pcs (*p* < 0.05). This resulted in a significant difference in pcs between the two groups after 12 months (*p* < 0.001, Table 4).

Baseline mcs values were significantly different between the two groups (*p* = 0.010) with patients in the PRI group having a higher baseline value. Although no significant change in the baseline value was noted 12 months after the intervention in patients in the PRI group, significant deterioration in patients in the FJI group was noted (*p* < 0.001, Table 4). This resulted in worsened mcs in the FJI group at the 12-month follow-up compared to the PRI group (*p* = 0.010).

Table 5 shows the results of patient satisfaction according to the MacNab criteria. Clear differences were noted between the groups in favor of the PRI group. A total of 92% of PRI patients (vs. 84% of FJI patients) reported excellent (complete relief of pain) or good (major relief of pain) results on the first day postintervention. Consecutively, 73% (vs. 48%) of patients had excellent or good results after six weeks, 55% (vs. 27%) after three months, 53% (vs. 14%) after six months, and 51% (vs. 26%) after 12 months.

### 3.5. Adverse Events

No differences between the groups were observed regarding the side effects after CT-based injection therapy. A total of 29 patients (15%) reported slightly transient and self-limiting side effects (1–4 h). These effects included initially increased low back pain (eight patients); numbness in the leg (ten patients); headache (seven patients); mild allergy, including redness of the face (three patients); and heartburn (one patient). Due to the self-limiting course of the side effects, no additional drug administration was necessary that could affect the outcome of the infiltration therapy. No serious adverse events were reported during the 12-month observation period.

## 4. Discussion

To the best of our knowledge, this study is the first to prospectively assess a direct comparison of the therapeutic value of infiltration therapies (FJI vs. PRI) in chronic complaints, under everyday clinical conditions. The study demonstrated that PRI showed a durable therapeutic effectiveness compared to FJI. Although a clinically meaningful pain reduction in back pain and possibly leg pain (MCID: NRS) was no longer detectable beyond three months after FJI, patients who received a PRI reported a clinically significant reduction in leg pain (LEP) and (if present) low back pain (LBP) over the entire period of the follow-up. The same applies to pain-associated disability in everyday life (MCID: ODI). Significant improvement in the physical health-related quality of life (SF-36: pcs) was noted in both groups, but patients who received a PRI benefited significantly more from infiltration therapy. The present results also reflect higher patient satisfaction in the PRI group.

The study demonstrates the dependence of the effectiveness of structural infiltration with local anesthetics and steroids. While a chronic neural affection seems to respond very well to the local application of the medication, arthrosis of the facet joints can only be affected to a limited extent by local therapy. The slight effect on arthritically altered joints does not seem surprising as this was also observed in other joints, such as the knee or hip joint [25,26]. The mechanism of action of a PRI with a local anesthetic and/or a steroid is probably based on neural blocking, which changes the reflex mechanism of the self-sustaining activity of the efferent fibers of the neurons and the pattern of the central neurons, thus interrupting nociceptive activity [27,28]. This pain modulation of the nerve tissue could explain the observed superior and longer-term effects of PRI despite the chronic pain characteristic of neuropathic pain. In addition, the decongestant effect of a steroid covering the affected nerve root is also observed; thus, a lower compression effect of the surrounding tissue on the nerve root is conceivable [29]. Furthermore, the natural course of both pathologies must be compared given that arthrosis typically progresses for more than a year, whereas compressed neural tissue, for example, after herniated discs, shows a clear tendency to recover even without therapeutic intervention [30,31]. Interestingly, we also found an effect of facet infiltration on leg pain and of nerve root infiltration on back pain. This controversy can be explained by accompanying muscle tone changes and functional complaints, such as sacroiliac joint disorders. Although not examined in direct comparison to the PRI, previous studies also observed a short-term pain-relieving effect of FJI in facet joint syndrome [32,33]. However, other studies examining the medium- to long-term effects of FJI on pain and function show divergent results [34,35,36]. The long-term relief of LBP after intra-articular steroid injections was between 18% and 63% in uncontrolled studies [37,38]. In controlled studies, the results are also inconsistent in the literature, and often no established scores were used to describe physical function [39,40]. Similar to the results of this study, Kawu et al. did not report any significant functional improvement, whereas Celik et al. indicated a significant improvement in function after six months [36,41]. However, significantly younger patients were included in the latter study, and radiologically proven facet joint arthropathy served as an exclusion criterion.

Clinically meaningful short- and long-term pain reduction after a PRI was demonstrated in other studies, which investigated the outcome of nerve root infiltration with radicular symptoms [42]. The authors reported an average pain reduction after root infiltration of 64–81% and functional improvement of 60–63%. Furthermore, Karpinnen et al. studied patients with LEP due to bony or discogenic stenosis who received a transforaminal epidural injection with an LA and steroid [39]. Analogous to the results of the present work, a significant improvement in pain (VAS) and function (ODI) was observed at the 12-month follow-up. However, no differences were noted in the placebo group, and Carette et al. found similar results regarding pain relief and functional improvement for steroid injection compared to placebo in regard to herniated discs [42]. The outcome of both studies underlines the potential of the natural course of neural convalescence with acute nerve compression, explaining the different durations of action of the FJI and PRI procedures. However, this notion must be compared to the fact that in the case of a herniated disc or spinal stenosis, infiltration of the nerve root with a local anesthetic and a steroid can prevent surgery by up to 71% (vs. 33% for the placebo group) [40]. This finding may be due to the positive effect in the acute and particularly painful phases, but only patients with chronic complaints were included in the present study. Hence, effectiveness in chronic persistent pain can be expected.

The presented study is not without limitations. First, a steroid was not applied to all patients, so the local drug composition was not completely uniform. However, the vast majority had steroid infiltrate. Based on the study design, effects attributed to the steroid versus the local anesthetic drug remain unclear. In this regard, individual studies show no major differences between local anesthetics and an LA/steroid mixture [2]. Second, because no contrast agent was used, we cannot completely exclude epidural spread, especially in the PRI group. However, the small volume applied, and the CT-guided needle position secured the selective nerve root block. However, it is not possible to determine exactly to what extent there was an allergic reaction or vascular passage in the presence of side effects. Another point is that the procedures were not compared to a placebo group. The comparison of the therapeutic effect of a PRI or FJI compared to a placebo group has already been investigated in numerous prospective studies [34,35,39,40]. However, the focus of this work was on comparing the individual PRI and FJI procedures. Therefore, a placebo group was not included.

Ultimately, there are also different infiltration techniques for the pathologies described (e.g., medial branch block vs. intraarticular infiltration vs. periarticular infiltration in patients with LBP or transforaminal vs. caudal vs. interlaminar approach in patients with LEP). Therefore, only conclusions about the techniques used can be drawn from the present study. Alternative forms of administration and application could show different durations. Thus, different effects could be observed depending on the approach used, which subsequently limits the comparability of the present work with other outcome studies that use alternative infiltration techniques.

## 5. Conclusions

We demonstrated that chronic back or leg pain, even when performed within a standardized setting, does not always respond equivalently to lumbar spinal needle intervention. Rather, the underlying pathology (facet joint arthritis vs. radiculopathy) seems to play a crucial role regarding therapeutic efficacy.

Based on the available results, CT-based PRI represents a suitable and easy method to provide long-acting therapy to patients with chronic radicular pain and associated LBP. Additionally, based on our study’s clinical results, infiltration of facet joints holds no notably durable therapeutic value and may be used as a diagnostic tool only to secure the potential cause of the complaint. Alternative forms of therapy, such as facet joint radiofrequency denervation, surgical procedures, and multimodal concepts, should be considered in this regard. 

## Figures and Tables

**Table 1:** Baseline demographic and clinical characteristics.

Groups (*n* = 196)		FJI Group (*n* = 87)	PRI Group (*n* = 109)	*p* Value
Gender	Men	38% (33)	44% (48)	0.388 *
	Woman	62% (54)	56% (61)	
Age [yrs]	Mean ± SD	66.2 ± 12.5	64.2 ± 11.6	0.248 ^†^
Weight [kg]	Mean ± SD	83.3 ± 16.7	83.5 ± 15.9	0.935 ^†^
Height [m]	Mean ± SD	1.7 ± 0.1	1.7 ± 0.1	0.254 ^†^
BMI [kg/m^2^]	Mean ± SD	28.8 ± 6.0	28.7 ± 5.1	0.883 ^†^
Numeric rating scale (0–10) ^‡^	Mean ± SD	6.9 ± 1.2	7.1 ± 1.3	0.402 ^†^
Oswestry Disability Index [%] (0–100)	Mean ± SD	44.4 ± 13.2	47.3 ± 17.0	0.177 ^†^
Grading of facet joint arthritis	Grade 3			n.a.
(Fujiwara)	Grade 4			
Grading of nerve root compression	Moderate	55% (48)	56% (61)	n.a.
	Severe	45% (39)	44% (48)	

* *p*-values from Fisher’s exact test; ^†^ *p*-values from Student’s *t*-test; ^‡^ numerical rating scale (NRS)overall including back and leg pain; SD—single standard deviation.

**Table 2:** Comparison of Numeric Pain Rating Scale (overall) and Oswestry Disability Index score between groups FJI and PRI over time.

Time	Numeric Pain Rating Scale		Oswestry Disability Index	
	FJI Group (87)	PRI Group (109)	FJI Group (87)	PRI Group (109)
	Mean ± SD	Mean ± SD	Mean ± SD	Mean ± SD
Baseline	6.9 ± 1.2	7.1 ± 1.3	44.4 ± 13.2	47.3 ± 17.0
After intervention	1.8 * ± 1.8	1.6 * ± 1.7	-	-
6 weeks ^†^	4.4 * ± 2.1	3.4 * ± 1.9	31.7 * ± 14.5	25.8 * ± 15.1
3 months ^†^	5.6 * ± 1.6	3.8 * ± 2.1	37.1 * ± 11.9	26.6 * ± 14.9
6 months ^†^	5.6 * ± 1.7	3.7 * ± 2.2	35.8 * ± 12.1	25.8 * ± 15.0
12 months ^†^	6.2 * ± 1.8	4.4 * ± 2.5	37.8 * ± 13.4	25.6 * ± 15.1
ptreatment	<0.001		<0.001	
ptime	<0.001		<0.001	
ptreatment × time	<0.001		<0.001	

*p*-values from 2-sided 2-way ANOVA for repeated measures; * indicates significant improvement in posthoc tests in comparison to baseline values (*p* < 0.001); ^†^ indicates a significant difference in posthoc tests between the means of NRS and ODI of the 2 groups at the specified time (*p* < 0.01); ODI—Oswestry Disability Index, SD—single standard deviation.

**Table 3:** Comparison of Numeric Pain Rating Scale for back pain and leg pain between the groups over time.

Time	Numeric Pain Rating Scale (Back)		Numeric Pain Rating Scale (Leg)	
	FJI Group (87)	PRI Group (109)	FJI Group (87)	PRI Group (109)
	Mean ± SD	Mean ± SD	Mean ± SD	Mean ± SD
Baseline ^†‡^	6.9 ± 1.7	5.7 ± 1.7	4.2 ± 2.8	7.2 ± 1.7
After intervention ^†^	2.4 * ± 1.6	1.3 * ± 1.4	1.2 * ± 1.7	1.8 * ± 1.4
6 weeks ^†‡^	4.6 * ± 2.3	2.6 * ± 2.1	2.6 * ± 2.5	3.5 * ± 2.3
3 months ^†^	5.7 * ± 1.9	2.9 * ± 2.3	3.4 * ± 2.5	3.9 * ± 2.6
6 months ^†^	5.7 * ± 2.0	2.8 * ± 2.4	3.4 * ± 2.5	3.6 * ± 2.6
12 months ^†^	6.0 * ± 1.9	3.6 * ± 2.6	4.5 ± 2.5	4.0 * ± 2.9
ptreatment	<0.001		0.009	
ptime	<0.001		<0.001	
ptreatment × time	<0.001		<0.001	

*p*-values from 2-sided 2-way ANOVA for repeated measures; * indicates significant improvement in posthoc tests in comparison to baseline values (*p* < 0.001); ^†^ indicates a significant difference in posthoc tests between the means of NRSback of the 2 groups at the specified time (*p* < 0.05); ^‡^ indicates a significant difference in posthoc tests between the means of NRSleg of the 2 groups at the specified time (*p* < 0.05) SD—single standard deviation.

**Table 4:** Comparison of SF-36—physical and mental health summary scale (pcs + mcs) between the groups over time.

Time	FJI Group (87)	PRI Group (109)	
	Mean ± SD	Mean ± SD	*p*-Value ^†^
	SF 36 (pcs)		
Baseline	31.1 ± 6.5	32.6 ± 7.4	0.120
12 months	30.3 ± 9.4 *	35.6 ± 7.2 *	<0.001
	SF 36 (mcs)		
Baseline	44.5 ± 8.3	47.8 ± 10.0	0.010
12 months	43.3 ± 9.0 *	48.3 ± 10.5	0.018

* *p*-values from paired samples Wilcoxon test indicates significant difference to baseline values within the group (*p* < 0.05); ^†^ *p*-values from Student’s *t*-test between the groups; SD—single standard deviation.

**Table 5:** Patients’ satisfaction with infiltration therapy according to MacNab’s-criteria.

		FJI Group (87)	PRI Group (109)	
Time	MacNab	Patients Treated	Patients Treated	*p*-Value
		n (%)	n (%)	
After intervention	Poor	3 (3)	0 (0)	0.007 *
	Fair	11 (13)	9 (8)	
	Good	42 (48)	37 (34)	
	Excellent	31 (36)	63 (58)	
6 weeks	Poor	11 (13)	2 (2)	<0.001 *
	Fair	34 (39)	28 (26)	
	Good	29 (33)	39 (36)	
	Excellent	13 (15)	40 (37)	
3 months	Poor	22 (25)	9 (8)	<0.001 *
	Fair	41 (47)	40 (37)	
	Good	12 (14)	32 (29)	
	Excellent	11 (13)	28 (26)	
6 months	Poor	35 (40)	22 (20)	<0.001 *
	Fair	40 (46)	29 (27)	
	Good	8 (9)	31 (28)	
	Excellent	4 (5)	27 (25)	
12 months	Poor	26 (30)	10 (9)	<0.001 *
	Fair	38 (44)	44 (40)	
	Good	15 (17)	28 (26)	
	Excellent	8 (9)	27 (25)	

* *p*-values from Fisher’s exact test indicate significant difference in patients’ satisfaction between the 2 groups.

## Data Availability

Not applicable.
